# Automated Cell Counting in CSF Diagnostics Revisited—Friend or Foe?

**DOI:** 10.3390/diagnostics15101202

**Published:** 2025-05-09

**Authors:** Axel Haarmann, Jörg Schubert, Udo Steigerwald, Michael K. Schuhmann

**Affiliations:** 1 Department of Neurology, University Hospital Würzburg, 97080 Würzburg, Germany; 2 Division of Laboratory Medicine, University Hospital Würzburg, 97080 Würzburg, Germany

**Keywords:** cerebrospinal fluid, Sysmex XN-9000, Fuchs–Rosenthal counting chamber, automated cell counting, CSF diagnostics

## Abstract

**Background/Objectives:** The gold standard for cerebrospinal fluid leukocyte counting is manual counting in a Fuchs–Rosenthal chamber. Recent advances in automated body-fluid-counting systems, offering a time- and labor-saving solution, are challenging this dogma. Yet, the equivalence of diagnostic accuracy is still debated in the community. **Methods:** We compared manual and automated cell counting of cerebrospinal fluid samples of lumbar punctures and extraventricular drains with both low and high leukocyte counts, shedding light on the variability of results between man and machine. **Results:** Automated and manual cell counting showed a strong correlation across all samples, particularly in the subgroup of patients with fewer than 20 cells/µl, where outliers could become especially clinically relevant. **Conclusions:** We found the automated counting system to be highly accurate and not lacking in diagnostic sensitivity even at low cell counts, making it a powerful tool when used in the right clinical setting.

## 1. Introduction

Cerebrospinal fluid (CSF) analysis is an essential backbone of the neurological diagnostic armamentarium. As a sensitive marker of intrathecal inflammation, the leukocyte count in particular is an important time-critical tool in acute diagnosis as well as in monitoring response to therapy. Since the beginning of the 20th century, the gold standard for counting CSF leukocytes has been manual counting in a Fuchs–Rosenthal counting chamber [1]. Automated cell-counting systems, well established in blood testing, are increasingly used in the analysis of many other body fluids. As cells pass through the detection system in an electrolyte solution, these machines use a combination of impedance-based counting and light-scattering techniques to quantify erythrocytes and platelets and identify different leukocyte types. Forward light scatter correlates with cell size, and side scatter correlates with granularity, providing information on cell structure and size. This allows for differentiation between mononuclear cells (lymphocytes, monocytes) and polymorphnuclear cells (such as neutrophils, eosinophils, and basophils) [2]. To date, automated CSF cell counting has only played a minor role in centers specializing in CSF diagnostics [3,4]. This is mainly due to a perceived lack of sensitivity at low event rates in both healthy and altered CSF [5,6,7,8]. On the contrary, these systems have the advantage of high availability and require less specialized staff to provide 24/7 diagnostics [9]. This is also important given the need for immediate processing of CSF samples, which can rapidly lose their diagnostic value if transport, storage, and analysis exceed a 2 h time frame [10]. For clinical practice, it is therefore necessary to clarify whether rapid automated counting is actually inferior to manual counting in all clinical scenarios (spinal tap, extraventricular drains, pure leukocytosis, and hemorrhagic CSF).

## 2. Materials and Methods

Between 25 July 2024 and 6 September 2024, we compared 119 CSF samples obtained as part of routine clinical examinations. These samples included 64 from lumbar punctures (LP) and 55 from an external ventricular drain (EVD). Samples (from the identical tube) were simultaneously analyzed (within 60 min), either by manual unstained counting in the Fuchs–Rosenthal chamber or by a Sysmex XN-9000 (Norderstedt, Germany) with body fluid mode. For manual counting, 20 µL of native CSF was added to the Fuchs–Rosenthal chamber and examined by an experienced technician using a Leica DM4B microscope (Wetzlar, Germany). If necessary, CSF was diluted with 0.9% NaCl (Fresenius Kabi, Bad Homburg, Germany) or with Türk’s solution (Sigma-Aldrich, Taufkirchen, Germany) prior to analysis.

For automated cell counting on the XN-9000 system, the measurement channel was first changed to body fluids mode. After flushing the system and carrying out a background measurement, it was ensured that there were no particles in the measurement channel that could lead to interference with the body fluids, which are generally low in cell counts. The measurement required 160 microlitres of CSF, of which 80 microlitres were used to measure the cell count.

The analysis was approved by the local ethics committee of the University of Würzburg, Germany (reference No 2024091801; 2024). Statistical testing was performed with GraphPad Prism (version 10.0, Boston, MA, USA).

## 3. Results

**Figure 1:**
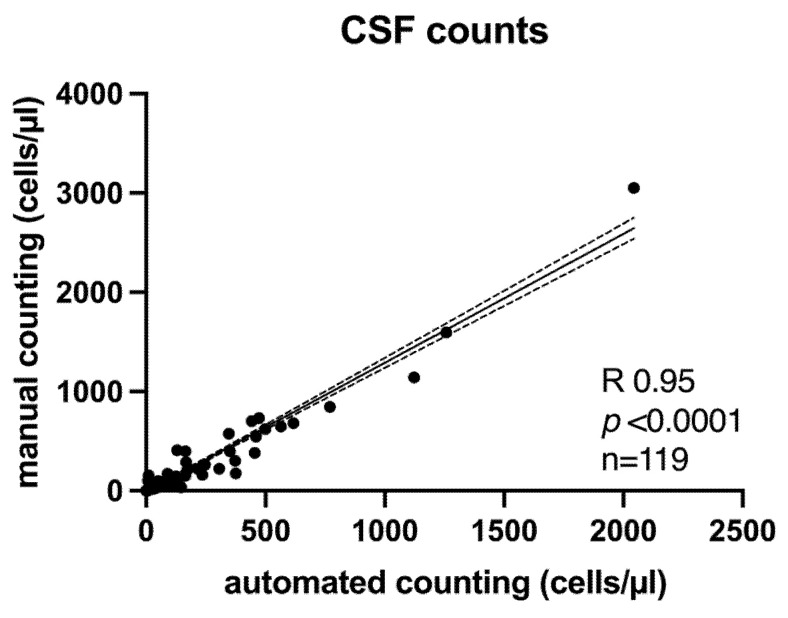
High correlation of CSF leukocyte counts assessed with manual and automated counting. Linear regression analysis with plot of the 95% confidence interval (dotted lines).

Overall, all samples (*n* = 119; LP = 64; EVD = 55) showed a high correlation of both counting methods (Figure 1) (R 0.95, *p* < 0.0001). There was a slight increase in deviation with increasing cell count but without systematic bias and within a range that did not affect clinical decisions.

**Figure 2:**
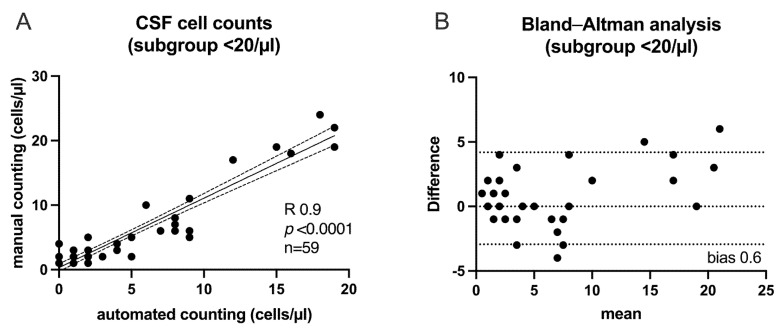
Subgroup of patients with low CSF cell counts (<20 cells/µL). Close correlation of manual and automated cell counts. Linear regression analysis with plot of the 95% confidence interval (dotted lines) (**A**). Bland–Altman analysis of the same collective to evaluate the agreement between the two different methods of measurement (**B**). The scattering of the differences around 0 indicates that there is no consistent difference between the methods. Additionally, most differences fall within the 95% limits of agreement suggesting interchangeability.

Given the higher diagnostic impact, particularly in influencing treatment decisions such as whether to initiate anti-infective treatment, we then focused on a sub-group of patients with a cell count of less than 20/µL (*n* = 59; LP = 51; EVD = 8), showing the same strong relationship between both techniques (Figure 2A) (R 0.9, *p* < 0.0001). To evaluate how well the two different measurement methods agree with each other, we used a Bland–Altman plot (Figure 2B). We were able to rule out systematic bias and show good consistency between the two techniques, suggesting that both methods can be used interchangeably.

Of the 59 patients, the clinical threshold of 5 cells/µL was crossed in 2 pairs (2 vs. 5 and 5 vs. 2 cells). Yet, this diagnostic uncertainty cannot be attributed to any of the methods and underlines why an evaluation in the clinical context is necessary. The detailed data are presented in Table 1.

To validate the reliability of the automated counting, we also performed a dilution series (Supplementary Materials, Figure S1).

## 4. Discussion

The need for rapid analysis and skilled personnel for manual cell counting continues to drive efforts to automate CSF analysis. For example, colleagues have recently introduced a new method using a microchip-based automated image analysis device for this purpose [11]. In response to this clinical need, we examined CSF samples using the Sysmex system XN-9000 with body fluid mode, which is already used in our laboratory workflow for hematology diagnostics. We show a representative dataset of comparative analysis of both LP and EVD CSF samples that yield comparable results irrespective of manual or automated cell counting. Given the important role of standardized pre-analytical handling, particularly in CSF diagnostics, the analysis was performed from CSF of the same collecting tube within 60 min.

Looking at the two samples that differed enough to cross a diagnostic border (2->5; 5->2; bold in Table 1), achieving a concordance rate of >98%, the difference was of borderline clinical relevance. We decided to include EVD samples in our analysis because we believe that automated cell counting can excel in this area. In patients with subarachnoidal hemorrhage for example, CSF samples are often heavily contaminated with blood and require significant dilution for manual counting, which can increase the potential for error. In this context, the key diagnostic parameter is the trend in cell count over time not detailed cell morphology [12]. Simple longitudinal leukocyte and erythrocyte counts (combined with glucose and lactat levels) are also sufficient to monitor the risk of ventriculitis [13]. Automated cell counting also has the advantage of providing rapid differentiation of leukocytes into clinically relevant mononuclear and polymorphnuclear cells, which is more time-consuming manually and requires staff experienced in differentiating stained cells. Although this may be sufficient for most time-critical decisions, one should keep in mind that automated differentiation provides only a rough picture of the situation. In particular, automated CSF cell counting is of limited diagnostic value when morphological assessment is required, for example, to detect malignant cells or to identify specific cell types, such as erythrophages or siderophages [14]. This also applies to blasts in suspected lymphomatous meningitis or plasma cells in certain neuroinflammatory conditions. In addition, CSF may be contaminated by chrondroblasts or bone marrow cells that are not correctly identified in automated analysis.

Although markedly elevated leukocyte counts and a high proportion of polymorphonuclear cells should already raise strong suspicion for bacterial meningitis, manual microscopy offers the critical advantage in such cases of potentially enabling direct detection of bacteria. Thus, in these scenarios, automation may fail to capture subtle but diagnostically important features, making manual microscopy essential for accurate evaluation.

These limitations can be addressed through well-structured clinical workflows that include routine cytospin preparation. While particularly relevant for patients with suspected malignancy or subarachnoid hemorrhage, we recommend this practice routinely, as lumbar punctures are often performed early in the diagnostic process, when clinical assessment may still evolve. Routine cytospin preparation helps avoid repeat punctures, minimizing patient burden while preserving the option for retrospective morphological evaluation.

Furthermore, the interpretation of automated counts requires a plausibility check by the physician. Here, modern automated counters with body fluid modes can support diagnostics by detecting cells with markedly increased autofluorescence, a potential indicator of malignancy typically linked to large nuclei [15]. While this method lacks the sensitivity and specificity of microscopy, such findings should prompt mandatory manual review to confirm or rule out the presence of malignant cells [16].

In general, caution is warranted, as automated classification of cellular elements in CSF carries an inherent risk of misinterpretation, especially in the absence of morphological validation. The core issue is that automated analyzers rely on the above mentioned physical and biochemical surrogates, such as cell size, impedance, or fluorescence intensity, to identify and classify particles. This creates a diagnostic black box, where the system may report numerical values without reliably distinguishing true cells from artifacts. Strongly fluorescent particles may be reported as highly fluorescence cells, but this label cannot distinguish malignant cells from non-cellular elements like protein aggregates or debris. This highlights how reliance on automated measurements can lead to inaccurate conclusions if the results are interpreted without considering the underlying technical limitations. Thus, while results of automated counters can be diagnostically useful, they require cautious interpretation and confirmation by manual microscopy.

## 5. Conclusions

In summary, automated cell counting serves as a reliable and complementary diagnostic tool in the modern CSF laboratory. However, it cannot replace hands-on microscopy as soon as detailed cytological evaluation, such as identifying malignant cells, is required. Thus, its use is limited more by the clinician’s ability to select the appropriate method and critically interpret the results than by technical feasibility or reliability.

## Figures and Tables

**Table 1:** Results of 59 CSF samples in the diagnostic critical range of up to 20 leukocytes/µL.

Material	Leukocytes (Automated)	Leukocytes (Manual)	Erythrocytes (Automated)	Erythrocytes (Manual)
LP	0	1	<500	<1
LP	0	1	<500	<1
LP	0	1	<500	<1
LP	0	1	<500	<1
LP	0	4	<500	<1
LP	0	1	<500	<1
LP	0	2	<500	<1
LP	1	1	<500	9
LP	1	3	<500	17
LP	1	1	<500	<1
LP	1	1	<500	124
LP	1	2	<500	<1
LP	1	3	<500	8
LP	1	1	<500	<1
LP	1	1	<500	<1
LP	1	2	<500	<1
LP	1	1	<500	<1
LP	1	3	<500	15
LP	1	1	<500	13
LP	1	1	<500	<1
LP	1	1	<500	<1
LP	1	3	<500	8
LP	1	2	<500	1
LP	1	2	<500	2
LP	1	3	<500	<1
LP	1	1	<500	1
LP	1	1	<500	1
LP	1	1	<500	<1
LP	1	1	<500	<1
LP	1	2	<500	2
LP	2	1	<500	n.d.
LP	2	1	<500	<1
LP	2	1	<500	<1
LP	2	2	<500	<1
***LP***	***2***	***5***	***<500***	***<1***
LP	2	3	<500	<1
LP	2	2	<500	2
LP	2	2	<500	1
LP	3	2	<500	14
EVD	4	4	4000	3093
LP	4	3	<500	<1
LP	4	4	<500	25
LP	5	5	<500	1
***LP***	***5***	***2***	***<500***	***2***
LP	6	10	<500	<1
LP	7	6	500–1000	<1
EVD	8	8	7000	5200
EVD	8	6	<500	84
LP	8	8	<500	64
LP	8	7	<500	<1
EVD	9	11	11,000	6683
EVD	9	5	1000	470
LP	9	6	<500	177
LP	12	17	<500	6
LP	15	19	<500	<1
LP	16	18	<500	<1
EVD	18	24	93,000	86,240
EVD	19	19	2000	2260
EVD	19	22	14,000	12,100

EVD: extraventricular drain, LP: lumbar puncture, n.d.: not determined.

## Data Availability

The datasets used and/or analyzed during the current study are available from the corresponding author on reasonable request.

## Supplementary Materials

The following supporting information can be downloaded at https://www.mdpi.com/article/10.3390/diagnostics15101202/s1: Figure S1: Serial dilution of a CSF sample measured in an automated cell counter.

## References

[1] Isenmann S., Strik H., Wick M., Gross C.C. (2017). Liquorzytologie: Methoden und Möglichkeiten. Fortschr. Neurol. Psychiatr..

[2] Alcaide Martin M.J., Altimira Queral L., Sahuquillo Frias L., Valina Amado L., Merino A., Garcia de Guadiana-Romualdo L. (2021). Automated cell count in body fluids: A review. Adv. Lab. Med..

[3] Aguadero V., Cano-Corres R., Berlanga E., Torra M. (2018). Evaluation of biological fluid analysis using the sysmex XN automatic hematology analyzer. Cytom. B Clin. Cytom..

[4] Cho J., Oh J., Lee S.G., Lee Y.H., Song J., Kim J.H. (2020). Performance Evaluation of Body Fluid Cellular Analysis Using the Beckman Coulter UniCel DxH 800, Sysmex XN-350, and UF-5000 Automated Cellular Analyzers. Ann. Lab. Med..

[5] Aune M.W., Becker J.L., Brugnara C., Canfield W., Dorfman D.M., Fiehn W., Fischer G., Fitzpatrick P., Flaming T.H., Henriksen H.K. (2004). Automated flow cytometric analysis of blood cells in cerebrospinal fluid: Analytic performance. Am. J. Clin. Pathol..

[6] Van Acker J.T., Delanghe J.R., Langlois M.R., Taes Y.E., De Buyzere M.L., Verstraete A.G. (2001). Automated flow cytometric analysis of cerebrospinal fluid. Clin. Chem..

[7] Wick M., Gross C.C., Tumani H., Wildemann B., Stangel M., On Behalf Of The German Society Of Csf D., Clinical Neurochemistry Dgln E.V. (2021). Automated Analysis of Cerebrospinal Fluid Cells Using Commercially Available Blood Cell Analysis Devices-A Critical Appraisal. Cells.

[8] Sandhaus L.M. (2005). Automated flow cytometric analysis of blood cells in cerebrospinal fluid. Am. J. Clin. Pathol..

[9] Zimmermann M., Ruprecht K., Kainzinger F., Heppner F.L., Weimann A. (2011). Automated vs. manual cerebrospinal fluid cell counts: A work and cost analysis comparing the Sysmex XE-5000 and the Fuchs-Rosenthal manual counting chamber. Int. J. Lab. Hematol..

[10] Tumani H., Petereit H.F., Gerritzen A., Gross C.C., Huss A., Isenmann S., Jesse S., Khalil M., Lewczuk P., Lewerenz J. (2020). S1 guidelines “lumbar puncture and cerebrospinal fluid analysis” (abridged and translated version). Neurol. Res. Pract..

[11] Park I., Choi M., Lee E., Park S., Jang W.S., Lim C.S., Ko S.Y. (2024). Evaluation of the Microscanner C3 for Automated Cell Counting in Cerebrospinal Fluid Analysis. Diagnostics.

[12] Zinganell A., Bsteh G., Di Pauli F., Rass V., Helbok R., Walde J., Deisenhammer F., Hegen H. (2022). Longitudinal ventricular cerebrospinal fluid profile in patients with spontaneous subarachnoid hemorrhage. Front. Neurol..

[13] Brooks M., Duong D., Shivapathasundram G., Sheridan M. (2022). Cerebrospinal fluid white cell count to red cell count ratio as a predictor of ventriculitis in patients with external ventricular drains. ANZ J. Surg..

[14] Strik H., Luthe H., Nagel I., Ehrlich B., Bahr M. (2005). Automated cerebrospinal fluid cytology: Limitations and reasonable applications. Anal. Quant. Cytol. Histol..

[15] Cho Y.U., Chi H.S., Park S.H., Jang S., Kim Y.J., Park C.J. (2015). Body fluid cellular analysis using the Sysmex XN-2000 automatic hematology analyzer: Focusing on malignant samples. Int. J. Lab. Hematol..

[16] Bonig L., Mohn N., Ahlbrecht J., Wurster U., Raab P., Puppe W., Suhs K.W., Stangel M., Skripuletz T., Schwenkenbecher P. (2019). Leptomeningeal Metastasis: The Role of Cerebrospinal Fluid Diagnostics. Front. Neurol..

