# Supplementary material for: Automated Cell Counting in CSF Diagnostics Revisited—Friend or Foe?
Source: Diagnostics (Basel). 2025 May 9;15(10):1202. doi: 10.3390/diagnostics15101202 (PMC12109621; doi:10.3390/diagnostics15101202)
Supplement: Supplementary file 1 [file diagnostics-15-01202-s001.zip › diagnostics-3594552-supplementary.pdf]

Supplementary Materials:

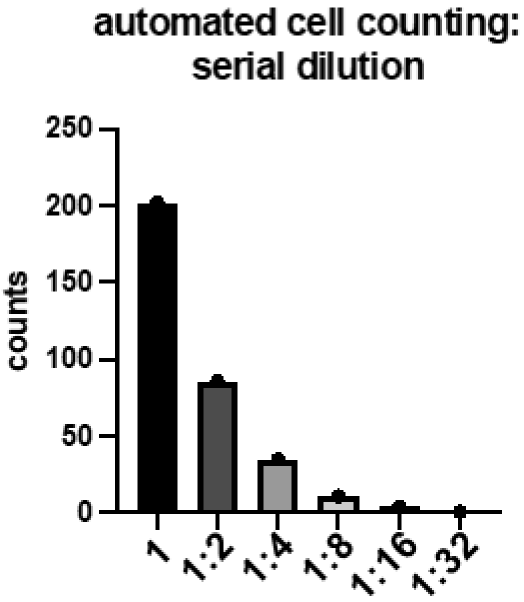

Figure S1. Serial dilution of a CSF sample measured in an automated cell counter.
